# Clinical relevance of circulating endocan and endoglin in excess body weight-associated endothelial dysfunction

**DOI:** 10.3389/fcvm.2026.1836882

**Published:** 2026-07-24

**Authors:** Charu Sharma, Abubaker Suliman, Ali Mohammed Aldhaheri, Saeed Mohammed Alnuaimi, Khaled Mohamed Alderei, Sania Mazin AlHamad, Javed Yasin Pathan, Elhadi H. Aburawi

**Affiliations:** 1 Department of Genetics and Genomics, College of Medicine and Health Sciences, United Arab Emirates University, Al Ain, United Arab Emirates; 2 Institute of Public Health, College of Medicine and Health Sciences, United Arab Emirates University, Al Ain, United Arab Emirates; 3 Department of Pediatrics, College of Medicine and Health Sciences, United Arab Emirates University, Al Ain, United Arab Emirates; 4 Department of Internal Medicine, College of Medicine and Health Sciences, United Arab Emirates University, Al Ain, United Arab Emirates

**Keywords:** cardiovascular disease, endocan, endoglin, endothelial dysfunction, inflammatory markers

## Abstract

**Introduction:**

Endothelial dysfunction is an early and pivotal event in the development of cardiovascular disease and a key mechanism linked to atherosclerotic plaque formation. Endocan and endoglin are emerging cardiometabolic markers and novel endothelial mediators that promote vascular smooth muscle cell proliferation and migration, contributing to intima-media thickening.

**Objectives:**

We aimed to assess endocan and soluble endoglin levels in young adults in the United Arab Emirates (UAE) and the associations among hyperglycemia, dyslipidemia, and endothelial dysfunction.

**Materials and methods:**

In this cross-sectional case-control study at UAE University, 182 young adults aged 18–22 years were classified as normal body weight (BW) (*n* = 61) or excess BW (*n* = 121) based on their body mass index. Anthropometry, systolic and diastolic blood pressures (SBP and DBP), inflammatory markers, the lipid profile, HbA1c, and plasma endocan and endoglin were measured. Multivariable linear regression analysis was performed to determine the associations between endoglin and log-transformed endocan and BW status and cardiometabolic variables, adjusting for age, sex, and family history of type 2 diabetes mellitus and hypertension.

**Results:**

Of the 182 participants, 88 (48%) were women, 121 (67%) had excess BW, and the mean (standard deviation) age was 19.96 years (1.79). Compared with the normal body weight group, the excess body weight group had significantly higher SBP, DBP, total cholesterol (TC), low-density lipoprotein cholesterol (LDL-C), high-sensitivity C-reactive protein (hs-CRP), tumor necrosis factor-*α*, interleukin-6, apolipoprotein B (Apo-B), and log-transformed endocan (all *p* ≤ 0.041), along with significantly lower high-density lipoprotein-cholesterol (*p* = 0.021) and apolipoprotein A (Apo-A) (*p* = 0.041) levels. In the adjusted models, endoglin was positively associated with excess BW, DBP, TC, and LDL-C, whereas log-transformed endocan was positively associated with excess BW, SBP, DBP, TC, LDL-C, Apo-B, and hs-CRP.

**Conclusions:**

Among these young adults, elevated endocan and endoglin levels correlated with excess body weight and adverse metabolic profiles. Prospective studies are needed to determine whether these biomarkers predict future cardiometabolic or vascular outcomes.

## Introduction

Endothelial dysfunction (ED) is a reversible condition characterized by diminished nitric oxide bioavailability and compromised endothelium-dependent vasodilation, leading to vascular constriction, inflammation, and decreased blood flow. As an early and crucial event in atherogenesis, ED increases vascular permeability, leukocyte adhesion, smooth muscle cell migration, and arterial remodeling, causing atherosclerotic vascular disease (1, 2).

Excessive adiposity facilitates ED via adipocyte hypertrophy, insulin resistance, oxidative stress, and dysregulated adipokine production. Elevated secretion of pro-inflammatory mediators, such as tumor necrosis factor-*α* (TNF-*α*), interleukin-6 (IL-6), and leptin, alongside decreased adiponectin levels, creates an imbalance that leads to low-grade systemic inflammation and endothelial activation. This creates a pathophysiological link between being overweight, early vascular damage, and a higher risk of cardiovascular disease (CVD) (3, 4). Thus, the endothelium-derived biomarkers endocan and endoglin provide greater insight into endothelial activation and vascular remodeling compared with other inflammatory biomarkers that indicate systemic inflammation (5).

Endocan is an endothelial proteoglycan that is increased by inflammatory cytokines, including TNF-*α* and IL-6. It is involved in activating the endothelium, causing inflammation and the formation of atherosclerosis (6). Endocan has also been linked to vascular diseases by facilitating the proliferation and migration of vascular smooth muscle cells, leading to neointimal hyperplasia and increased intima-media thickness, both of which are indicative of early atherosclerotic changes (7–9). Elevated levels of endocan have been reported in inflammatory disorders, atherosclerosis, and diabetic nephropathy (8, 10).

Endoglin is a homodimeric transmembrane glycoprotein that serves as a coreceptor for transforming growth factor-*β* (TGF-*β*) and is primarily expressed on vascular endothelial cells (11). It is crucial for angiogenesis, endothelial cell proliferation, and blood vessel remodeling. Membrane-bound endoglin facilitates endothelial proliferation and angiogenic signaling, but soluble endoglin, produced by proteolytic cleavage, disrupts TGF-*β* signaling and hinders endothelial migration and capillary formation, ultimately leading to vascular dysfunction. Elevated soluble endoglin levels have been reported in conditions such as preeclampsia (12, 13), diabetic nephropathy (14), hypertension (HTN), and type 2 diabetes mellitus (T2DM) with microvascular consequences, corroborating its role as a marker of endothelial dysfunction and obesity-related vascular disease (14, 15).

Due to lifestyle changes, there has been an increase in overweight and obesity among children and adolescents in the United Arab Emirates (UAE). Studies have reported that approximately 28% of school-aged children and teenagers are overweight or obese (16). This is largely due to a sedentary lifestyle, reduced physical activity, and eating greater amounts of processed foods that are high in calories. Childhood and adolescent obesity is clinically significant due to its correlation with insulin resistance, dyslipidemia, low-grade inflammation, and early vascular changes that may endure into adulthood, hence elevating lifetime cardiovascular disease risk (17).

The regional metabolic burden further emphasizes the necessity of early risk identification. The International Diabetes Federation has highlighted the increased burden of diabetes in the Middle East and North Africa. In 2024, nearly 85 million individuals were diagnosed with diabetes, and this is expected to rise to 163 million by 2050 (18). Adult data comprises the majority of national and regional statistics; however, children and adolescents with T2DM are becoming more common as obesity rates rise. In the UAE, where childhood obesity rates have been reported to surpass international norms in some age groups, advanced endothelium biomarkers may enhance early metabolic and vascular risk assessment in a multiethnic population (19, 20).

In early life, excess adiposity, dyslipidemia, hyperglycemia, and low-grade inflammation may precipitate vascular damage and confer enduring cardiovascular risk in later life. Therefore, assessing these biomarkers in young adults indicates early endothelial activation and remodeling, which may help avert progression to clinical CVD. This study aimed to investigate circulating endocan and endoglin levels in young adults with normal or excess body weight (BW) and further analyze their correlation with other cardiometabolic risk factors.

## Materials and methods

### Participants

This cross-sectional case-control study was conducted at the UAE University in Al Ain. The 18–22 age range was selected in accordance with established clinical guidelines (21, 22). Asymptomatic young adults aged 18–22 years were included and categorized into two groups based on body mass index (BMI). Participants with a BMI ≥ 25 kg/m^2^ were classified as having excess BW, while those with a BMI between 18 and 24.9 kg/m^2^ were considered to have normal BW. The exclusion criteria were the presence of any medical illness, a family history of premature CVD, clinical instability within the preceding year, and the use of regular medications or vitamin supplements. A family history of premature CVD was included in the exclusion criteria to reduce the confounding effect from a strong inherited high-risk CVD background in an exploratory biomarker study of otherwise asymptomatic young adults. However, family histories of diabetes mellitus and hypertension were included as covariates in the adjusted analysis (23).

### Anthropometric measurements

A trained research nurse used a validated questionnaire to collect data from young adults on their history of smoking; family histories of hypertension, diabetes mellitus, myocardial infarction; and parental consanguinity. Systolic and diastolic blood pressures (SBP and DBP) were measured using a calibrated Welch Allyn ProBP 3400 Series automated monitor (NY, USA), with the cuff size appropriately adjusted to arm circumference. The participants were allowed to rest for 5 min and then asked to sit with back support, feet flat on the floor, and arms resting on a table at heart level. The cuff was placed on their bare left upper arm, and their blood pressure was measured three times with 5 min rest intervals, with the average reading then recorded.

BW was measured to the nearest 0.1 kg using a digital scale, with the participants wearing light clothing. Height was measured without shoes to the nearest 0.1 cm using a stadiometer in a standing position.

### Blood collection and biomarker measurements

After an overnight fast, blood samples were collected, and plasma and serum were separated and processed for different laboratory tests. The samples were centrifuged at 4,000 rpm for 10 min, and the plasma and serum aliquots were stored at −80°C until further analysis.

### Endothelial dysfunction biomarker

Soluble endocan and endoglin levels were measured in processed plasma samples using commercially available enzyme-linked immunosorbent assay (ELISA) kits, following the manufacturers’ protocols. The ELISA kit that was used to measure endoglin levels was CD105/ENG, from MyBioSource (CA, USA; Cat. no.: MBS2601416) with a detection range of 10 ng/mL–0.156 ng/mL, sensitivity up to 0.05 ng/mL, intra-assay precision ≤ 8%, and inter-assay precision ≤ 12%. Endocan levels were analyzed using the Human Endothelial Cell Specific Molecule-1 (ESM-1) ELISA kit, manufactured by MyBioSource (CA, USA; Cat. no.: MBS009579), with a sensitivity of 5.0 pg/mL, a detection range of 31.2 pg/mL–1,000 pg/mL, and intra-assay coefficient of variation (CV) (%) and inter-assay CV (%) less than 15%.

### Inflammatory biomarkers

The IL-6 plasma concentration was detected using the Human IL-6 (Cat. no.: D6050; R&D Systems, NE, USA), with a sensitivity of < 0.626 pg/mL, a detection range between 3.13 and 300 pg/mL, an intra-assay precision of ∼3.0%–5.0% CV, and an inter-assay precision of ∼4.0%–7.0% CV. TNF-*α* was detected using Human TNF-*α* (Cat. no.: DTA00D, R&D Systems, NE, USA), with a sensitivity between 2.09 and 6.23 pg/mL. The detection range of this kit was 15.6–1,000 pg/mL, with an intra-assay precision of ∼4.5%–6.0% CV and an inter-assay precision of ∼5.5%–7.5% CV. The high-sensitivity C-reactive protein (hs-CRP) level was analyzed using an Integra 400 Plus automated analyzer (Roche Diagnostics, Mannheim, Germany) with a standardized kit.

### Dyslipidemia biomarkers

Biochemical analyses, including total cholesterol (TC), high-density lipoprotein cholesterol (HDL-C), low-density lipoprotein cholesterol (LDL-C), triglycerides (TG), apolipoprotein A (Apo-A), apolipoprotein B (Apo-B), and glycated hemoglobin (HbA1c), were conducted using an Integra 400 Plus automated analyzer (Roche Diagnostics, Mannheim, Germany) with standardized kits.

### Sample size calculation

The sample size was calculated using the formula for a continuous outcome as described by Bhardwaj et al. (24). Initial estimates were based on data from Atef et al. (25), who reported soluble endoglin concentrations of 5.40 ± 0.69 pg/mL in obese adults and 4.60 ± 0.71 pg/mL in healthy controls. These values yielded an expected mean difference of 0.80 and a pooled standard deviation (SD) of 0.70, corresponding to a standardized effect size (*d* = 1.14). With a two-sided significance level of *α* = 0.05 and 90% power, and anticipating small biomarker differences in our target population, we utilized a conservative standardized effect size of Cohen's *d* = 0.50 and upheld a 2:1 case–control ratio, yielding 126 cases and 63 controls.

### Statistical analysis

Descriptive statistics were computed for all variables. Categorical variables are summarized as frequencies and percentages, while continuous variables are reported as the mean ±  SD for normally distributed data or the median with the interquartile range (Q1, Q3) for non-normally distributed data. Because the endocan values were positively skewed, a logarithmic transformation was applied to reduce the influence of extreme values and better satisfy the assumptions of linear regression.

Group comparisons were conducted using an independent two-sample *t*-test or a Wilcoxon rank-sum test for continuous variables, and a Pearson's chi-squared test for categorical variables. Spearman's correlation coefficient was used to investigate the relationships between continuous variables. Univariable and multivariable linear regression models were constructed to assess the association between endoglin and log-transformed endocan and body weight status and cardiometabolic variables. The multivariable models were adjusted for age, sex, and family histories of T2DM and hypertension. Multicollinearity was assessed using the variance inflation factor (VIF). In all the adjusted models, the VIF values were low (all < 1.3), indicating no evidence of multicollinearity. A two-sided *p*-value <0.05 was considered statistically significant. All the statistical analyses were performed using R software (version 4.3.1).

## Results

A total of 182 participants were included in our analysis, 121 of whom (67%) had excess BW. Overall, the mean (SD) age was 19.96 (1.79) years, 88 participants (48%) were women, and approximately one-third of the cohort had a family history of HTN or T2DM. The overall mean (SD) and median (IQR) of log-transformed endocan and endoglin were 3.90 pg/mL (0.93) and 3.76 pg/mL (3.11, 4.54), and 1.35 ng/mL (0.52) and 1.19 ng/mL (1.03, 1.46), respectively (Table 1).

**Table 1:** Anthropometric, clinical, and biomarker characteristics of the participants according to body weight status.

Variable	Overall^a^ *N* = 182	Normal body weight^a^ *N* = 61	Excess body weight^a^ *N* = 121	*p*-Value^b^
Age (years)	19.96 (1.79)	19.89 (1.77)	19.99 (1.81)	0.700
Sex, female	88 (48%)	26 (43%)	62 (51%)	0.300
BMI (kg/m^2^)	29 (7)	22 (2)	32 (7)	**<0** **.** **001**
Family history of HTN	60 (34%)	20 (33%)	40 (34%)	0.900
Family history of DM	61 (35%)	16 (27%)	45 (39%)	0.110
SBP (mm Hg)	121 (12)	114 (9)	124 (13)	**<0**.**001**
DBP (mm Hg)	74 (8)	68 (5)	76 (8)	**<0**.**001**
TC (mmol/L)	4.22 (0.78)	4.19 (0.74)	4.24 (0.81)	0.700
HDL-C (mmol/L)	1.33 (0.40)	1.43 (0.40)	1.28 (0.39)	**0**.**021**
LDL-C (mmol/L)	2.85 (0.81)	2.67 (0.71)	2.94 (0.84)	**0**.**029**
TG (mmol/L)	1.10 (0.57)	1.01 (0.53)	1.14 (0.59)	0.200
HbA1c (%)	4.94 (0.62)	4.99 (0.45)	4.92 (0.69)	0.500
Apo-A (g/L)	1.44 (0.26)	1.49 (0.24)	1.41 (0.27)	**0**.**041**
Apo-B (g/L)	0.88 (0.24)	0.81 (0.21)	0.92 (0.24)	**0**.**003**
hs-CRP (mg/L)	3.0 (4.6)	1.2 (2.4)	3.9 (5.2)	**<0**.**001**
TNF-*α* (pg/mL)	4.05 (2.42)	3.03 (2.07)	4.56 (2.43)	**<0**.**001**
IL-6 (pg/mL)	2.51 (1.67)	1.83 (1.35)	2.84 (1.71)	**<0**.**001**
Log-transformed endocan (pg/mL)	**0.001**			
Mean (SD)	3.90 (0.93)	3.62 (0.89)	4.04 (0.92)	
Median (Q1, Q3)	3.76 (3.11, 4.54)	3.44 (3.03, 3.90)	4.06 (3.39, 4.65)	
Endoglin (ng/mL)	0.063			
Mean (SD)	1.35 (0.52)	1.20 (0.30)	1.42 (0.59)	
Median (Q1, Q3)	1.19 (1.03, 1.46)	1.13 (1.02, 1.33)	1.20 (1.03, 1.53)	

a Continuous variables are summarized using the mean (SD), except for endocan and endoglin, which are summarized using the mean (SD) and median (Q1, Q3).

b Continuous variables were compared using the independent two-sample *t*-test, whereas endocan and endoglin were assessed using the Wilcoxon rank-sum test. Categorical variables were compared using Pearson’s chi-squared test. *P*-values in bold indicate statistical significance at the 5% level.

IQR, interquartile range; SD, standard deviation; BMI, body mass index; HTN, hypertension; DM, diabetes mellitus; TC, total cholesterol; TG, triglycerides.

Compared with the normal BW group, participants with excess BW had significantly higher SBP, DBP, LDL-C, Apo-B, hs-CRP, TNF-*α*, IL-6, and log-transformed endocan and significantly lower HDL-C and Apo-A levels (all *p* < 0.05). Age, sex distribution, family history of HTN, family history of diabetes mellitus (DM), TC, TG, and HbA1c did not differ significantly between the two groups (Table 1).

**Figure 1:**
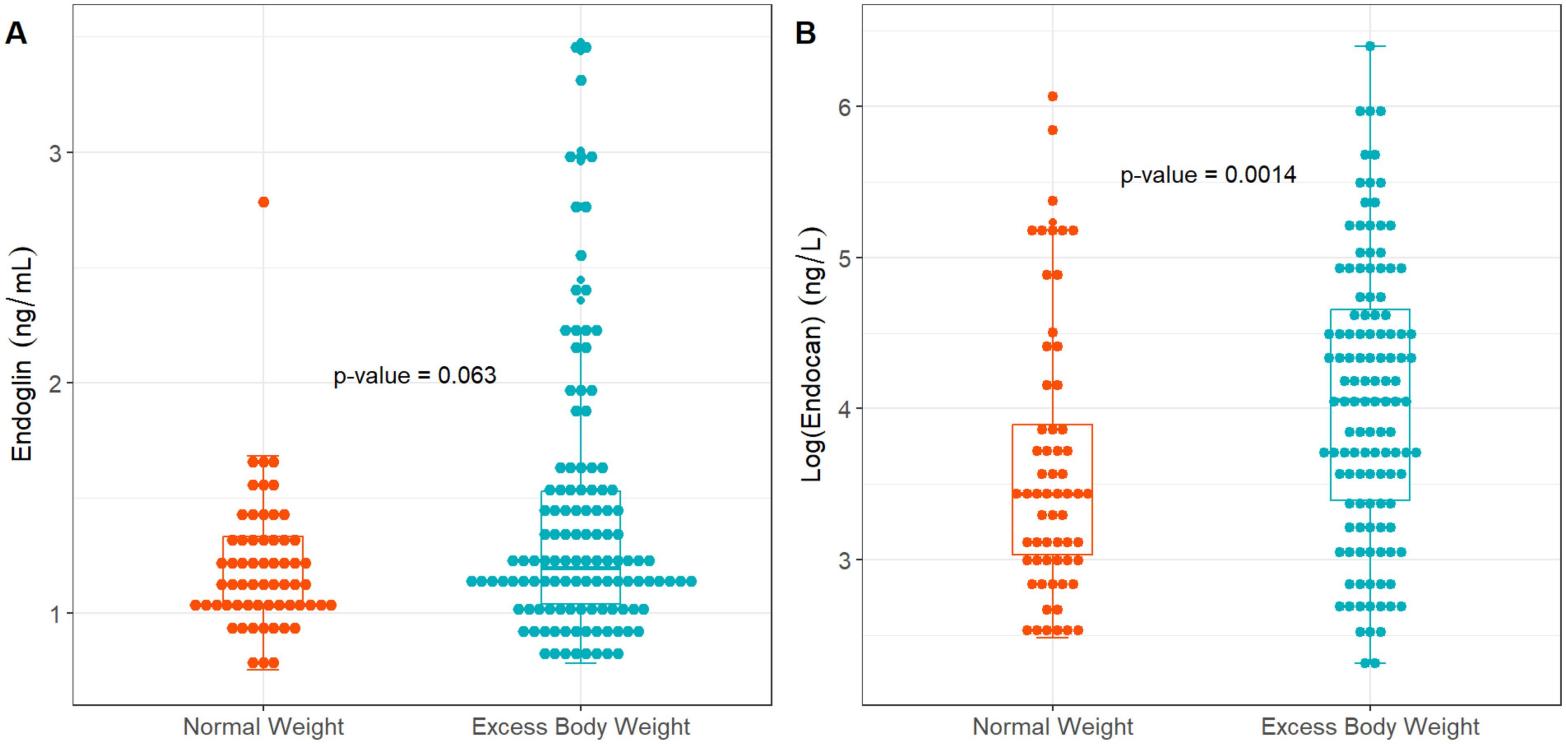
The differences between the normal weight and excess weight groups in terms of **(A)** endoglin and **(B)** endocan levels.

Figure 1 shows the differences in endocan and endoglin levels between the normal BW and excess BW groups. Endoglin levels tended to be higher in participants with excess BW; however, the difference did not reach statistical significance (*p* = 0.063). In contrast, the log-transformed endocan levels were significantly higher in the participants with excess body weight, consistent with greater endothelial activation (*p* = 0.0014).

Table 2 shows that log-transformed endocan levels had significant positive correlations with SBP and DBP in both the normal BW and excess BW groups. Endoglin levels were positively correlated with TC and TG. We did not observe a significant correlation between endocan and endoglin and age, HDL-C, LDL-C, HbA1c, Apo-A, Apo-B, hs-CRP, TNF-*α*, or IL-6.

**Table 2:** Spearman’s correlation coefficients for endoglin and log-transformed endocan levels and cardiometabolic variables in the normal BW and excess BW groups.

Variable	Endoglin		Log-transformed endocan	
	Excess BW	Normal BW	Excess BW	Normal BW
SBP (mmHg)	−0.045	0.362	0.216***	0.105***
DBP (mmHg)	0.057	−0.129	0.188**	−0.111**
TC (mmol/L)	0.222*	0.048*	0.136	0.118
TG (mmol/L)	0.090*	0.205*	−0.066	0.082

* *p* < 0.05; **p < 0.01; and ***p < 0.001.

A univariable linear regression analysis (Table 3) showed that excess body weight, DBP, TC, and LDL-C were significantly associated with both endoglin and log-transformed endocan levels, implying a metabolic and vascular link with these endothelial markers. In addition, log-transformed endocan levels showed a significant association with SBP, Apo-B, and hs-CRP.

**Table 3:** Univariable linear regression analyses of body weight status and cardiometabolic variables with endoglin and log-transformed endocan.

Variable	Endoglin *β* (95% CI)	Endoglin *p*-value	Log-transformed endocan *β* (95% CI)	Log-transformed endocan *p*-value
Body weight				
Normal BW	Ref		Ref	
Excess BW	0.22 (0.06, 0.38)	**0**.**007**	0.42 (0.14, 0.71)	**0**.**003**
SBP (mmHg)	0.00 (−0.00, 0.01)	0.581	0.02 (0.01, 0.03)	**0**.**002**
DBP (mmHg)	0.01 (0.00, 0.02)	**0**.**039**	0.02 (0.01, 0.04)	**0**.**009**
TC (mmol/L)	0.12 (0.02, 0.22)	**0**.**015**	0.23 (0.06, 0.40)	**0**.**008**
HDL-C (mmol/L)	−0.03 (−0.22, 0.17)	0.787	−0.12 (−0.46, 0.22)	0.494
LDL-C (mmol/L)	0.12 (0.03, 0.21)	**0**.**012**	0.20 (0.04, 0.37)	**0**.**016**
TG (mmol/L)	0.02 (−0.12, 0.15)	0.819	0.12 (−0.12, 0.36)	0.311
HbA1c (%)	−0.11 (−0.23, 0.01)	0.083	0.11 (−0.11, 0.33)	0.308
Apo-A (g/L)	−0.12 (−0.41, 0.17)	0.426	−0.13 (−0.65, 0.39)	0.631
Apo-B (g/L)	0.27 (−0.05, 0.59)	0.100	0.71 (0.15, 1.27)	**0**.**013**
hs-CRP (mg/L)	0.01 (−0.01, 0.02)	0.329	0.03 (0.00, 0.06)	**0**.**034**
TNF-*α* (pg/mL)	0.02 (−0.01, 0.05)	0.251	0.05 (−0.01, 0.11)	0.117
IL−6 (pg/mL)	0.03 (−0.02, 0.07)	0.307	0.05 (−0.04, 0.13)	0.294

*p*-values in bold indicate statistical significance at the 5% level.

In the multivariable linear regression model, models were adjusted for age, gender, and family histories of diabetes and hypertension. After adjustment, endoglin was independently associated with excess BMI [*β* (95% CI) = 0.26 (0.10, 0.42), *p* = 0.002], DBP [*β* (95% CI) = 0.01 (0.00, 0.02), *p* = 0.044], TC [*β* (95% CI) = 0.14 (0.04, 0.24), *p* = 0.006], and LDL-C [*β* (95% CI) = 0.14 (0.04, 0.23), *p* = 0.005]. Similarly, the log-transformed endocan levels showed an independent association with excess BMI [*β* (95% CI) = 0.47 (0.19, 0.76), *p* = 0.001], DBP [*β* (95% CI) = 0.02 (0.01, 0.04), *p* = 0.006], TC [*β* (95% CI) = 0.25 (0.07, 0.42), *p* = 0.007] and LDL-C [*β* (95% CI) = 0.22 (0.05, 0.39), *p* = 0.013]. In addition, log-transformed endocan levels were independently associated with Apo-B [*β* (95% CI) = 0.67 (0.10, 1.24), *p* = 0.022] and SBP [*β* (95% CI) = 0.02 (0.00, 0.03), *p* = 0.014] (Table 4).

**Table 4:** Multivariable linear regression analyses of body weight status and cardiometabolic variables with endoglin and log-transformed endocan, adjusted for age, sex, family history of diabetes, and family history of hypertension.

Variable	Endoglin		Log-transformed endocan	
	Beta (95% CI)	*p*-Value	Beta (95% CI)	*p*-Value
Body weight				
Normal BW	Ref		Ref	
Excess BW	0.26 (0.10, 0.42)	**0**.**002**	0.47 (0.19, 0.76)	**0**.**001**
SBP (mmHg)	0.00 (−0.01, 0.01)	0.607	0.02 (0.00, 0.03)	**0**.**014**
DBP (mmHg)	0.01 (0.00, 0.02)	**0**.**044**	0.02 (0.01, 0.04)	**0**.**006**
TC (mmol/L)	0.14 (0.04, 0.24)	**0**.**006**	0.25 (0.07, 0.42)	**0**.**007**
LDL-C (mmol/L)	0.14 (0.04, 0.23)	**0**.**005**	0.22 (0.05, 0.39)	**0**.**013**
HDL-C (mmol/L)	−0.00 (−0.21, 0.21)	0.984	0.02 (−0.35, 0.40)	0.898
TG (mmol/L)	0.03 (−0.11, 0.17)	0.711	0.10 (−0.15, 0.35)	0.428
HbA1C (%)	−0.11 (−0.24, 0.01)	0.076	0.08 (−0.14, 0.31)	0.474
Apo-A (g/L)	−0.07 (−0.37, 0.23)	0.642	0.03 (−0.50, 0.56)	0.914
Apo-B (g/L)	0.28 (−0.04, 0.60)	0.086	0.67 (0.10, 1.24)	**0**.**022**
hs-CRP (mg/L)	0.00 (−0.01, 0.02)	0.623	0.03 (−0.00, 0.06)	0.052
TNF-*α* (pg/mL)	0.01 (−0.02, 0.05)	0.461	0.04 (−0.02, 0.11)	0.169
IL-6 (pg/mL)	0.02 (−0.03, 0.07)	0.439	0.05 (−0.04, 0.14)	0.297

*p*-values in bold indicate statistical significance at the 5% level.

## Discussion

In this study, excess BW was associated with higher blood pressure (BP), a more atherogenic lipid profile (elevated LDL-C/Apo-B and reduced HDL-C/Apo-A), and elevated inflammatory and endothelial dysfunction markers (hs-CRP, TNF-*α*, IL-6, and log-transformed endocan). A normal BW, compared with excess BW, was associated with significantly elevated endocan levels and a trend toward higher endoglin levels. This suggests that endothelial activation/dysfunction is already detectable in young adults with excess weight, even when some conventional risk markers (e.g., HbA1c) are still normal. In the excess BW group, endocan behaved the same way as an inflammation-linked endothelial marker, as it rose with SBP/DBP and trended with inflammatory cytokines. Endoglin levels were comparatively uninformative in the excess BW group, but in the normal BW group, they were associated with SBP and atherogenic lipids, indicating that endoglin may capture vascular/lipid stress earlier in leaner individuals, whereas endocan becomes more responsive as adiposity and BP increase. As glycemic status (HbA1c) and TC were not different between the groups, these findings suggest that early cardiometabolic changes are driven more by blood pressure, lipid partitioning, and inflammation than by average glycemia in this cohort.

Our findings are broadly consistent with previous studies in UAE populations that reported that excess adiposity in children, adolescents, and young adults is accompanied by dyslipidemia, inflammation, and endothelial dysfunction. A study in obese UAE youth aged 18–22 showed significant correlations between ED markers and lipid profiles, as well as inflammatory and oxidative stress biomarkers (26). Another study in youth from the UAE with diabetes showed that subclinical inflammation and ED are frequent and observed an association with obesity and dyslipidemia, particularly low HDL-C (27). Another study on Emirati schoolchildren with overweight or obesity showed significant increases in inflammatory markers (hs-CRP, IL-6, and TNF-*α*), endothelial dysfunction markers (sICAM-1 and sVCAM-1), and atherogenic lipid changes (elevated TG and reduced HDL-C) compared with peers of normal weight. These findings demonstrate that even at primary-school ages, excess fat is associated with subclinical inflammation, dyslipidemia, endothelial stress, and liver function disturbances, supporting early lifestyle interventions and highlighting the childhood origins of cardiometabolic disease (28). Although these studies do not directly address future cardiovascular events, they do suggest a potential link between these biomarkers and cardiovascular risk factors.

Another study suggested that serum endocan levels are positively correlated with both traditional and non-traditional anthropometric indices in adults, with non-traditional indices such as the body adiposity index (BAI) and cardiometabolic index (CMI) showing independent associations. They used traditional indices (BMI, waist circumference, and waist-to-hip ratio) and non-traditional indices (BAI and CMI) in a univariate regression analysis, showing positive correlations between serum endocan levels and nearly all measured anthropometric parameters. This indicates that endocan can serve as a relevant biomarker for assessing cardiometabolic risk related to obesity and body composition in adults (29).

Another case-control study of UAE students (18–22 years) strengthened the evidence that endothelial perturbations are already detectable in early adulthood and cocluster with excess adiposity and cardiometabolic risk factors (30, 31). In our study, relative to normal BW peers, young adults with excess BW exhibited higher log-transformed endocan, more adverse hemodynamics and lipids (elevated SBP/DBP, elevated LDL-C, and decreased HDL-C), and a more inflammatory milieu (elevated hs-CRP, TNF-*α*, and IL-6). The multivariable analyses showed that endoglin was positively associated with excess BW, DBP, TC, and LDL-C, whereas log-transformed endocan was positively associated with excess BW, SBP, DBP, TC, LDL-C, and Apo-B. Moreover, male participants had higher concentrations of both biomarkers than female participants. In agreement with previous studies, our study also showed that endocan and endoglin track distinct dimensions of early vascular stress, namely inflammation (endocan) and dyslipidemia (endoglin), in a population otherwise considered clinically healthy (5).

The literature on endocan is not fully uniform across populations and metabolic phenotypes. Endocan is secreted by activated endothelium in response to pro-inflammatory cytokines and has been linked to atherogenic remodeling (8, 32). Reports of children and adolescents with obesity have shown higher circulating levels of endocan and links to subclinical atherosclerosis and fatty liver disease (9, 10). Adult cross-sectional data have also associated endocan with adverse anthropometric and metabolic indices (29). Endoglin is the soluble form of the TGF-*β* coreceptor endoglin and perturbs endothelial TGF-*β* signaling, impairing endothelial migration and angiogenic capacity (11, 33, 34). Elevated endoglin levels have been described across vascular pathologies, including hypertension and diabetes (15). Thus, low-grade inflammation and mild dysglycemia would be expected to increase one’s endocan levels, while lipid-induced endothelial stress would be reflected in one’s endoglin levels. This is consistent with our data and with the contemporary endothelium-centric model of atherogenesis (30).

Therefore, our findings should not be interpreted as a sequence of biomarkers or as a mechanistic study, and our data do not directly demonstrate that endoglin reflects vascular or lipid stress earlier in young adults of normal body weight or that endocan becomes more responsive as adiposity and blood pressure increase. These interpretations are better viewed as hypotheses for future research than as conclusions supported by the current analysis.

## Strengths and limitations

The strengths of this study include the narrow age range and the multivariable modeling that accounted for adiposity class and key cardiometabolic covariates. The limitations include its cross-sectional case-control design, limited fat distribution analysis, single-center sampling, and single-timepoint biomarkers. We could not make any predictions or draw any causal inferences. Vascular function endpoints (e.g., flow-mediated dilation, pulse-wave velocity, and intima-media thickness) were not assessed in the asymptomatic young adults. Consequently, incorporating such measures would strengthen the criterion validity and enable mediation analyses.

## Conclusion

In this study, elevated endocan and endoglin levels were observed in young adults with excess body weight and adverse cardiometabolic characteristics. Endoglin was correlated with adverse lipid parameters, while endocan was linked to unfavorable glycemic indices, suggesting differential roles in early metabolic dysregulation. These findings highlight the potential utility of these endothelial biomarkers in identifying subclinical vascular risk during young adulthood. Further longitudinal studies are warranted to determine their role in cardiometabolic disorders and early intervention strategies.

## Data Availability

The original contributions presented in the study are included in the article/Supplementary Material, further inquiries can be directed to the corresponding author.
